# Metabolic Regulation of “*Ca*. *Methylacidiphilum Fumariolicum*” SolV Cells Grown Under Different Nitrogen and Oxygen Limitations

**DOI:** 10.3389/fmicb.2012.00266

**Published:** 2012-07-25

**Authors:** Ahmad F. Khadem, Arjan Pol, Adam S. Wieczorek, Mike S. M. Jetten, Huub J. M. Op den Camp

**Affiliations:** ^1^Department of Microbiology, Institute of Water and Wetland Research, Radboud University NijmegenNijmegen, Netherlands

**Keywords:** *Methylacidiphilum*, methane, RNA-Seq, Verrucomicrobia, metabolic regulation, nitrogen, pMMO

## Abstract

Aerobic methanotrophic bacteria can use methane as their sole energy source. The discovery of “*Ca*. *Methylacidiphilum fumariolicum*” strain SolV and other verrucomicrobial methanotrophs has revealed that the ability of bacteria to oxidize CH_4_ is much more diverse than has previously been assumed in terms of ecology, phylogeny, and physiology. A remarkable characteristic of the methane-oxidizing Verrucomicrobia is their extremely acidophilic phenotype, growing even below pH 1. In this study we used RNA-Seq to analyze the metabolic regulation of “*Ca*. *M. fumariolicum*” SolV cells growing at μ_max_ in batch culture or under nitrogen fixing or oxygen limited conditions in chemostats, all at pH 2. The analysis showed that two of the three *pmoCAB* operons each encoding particulate methane monoxygenases were differentially expressed, probably regulated by the available oxygen. The hydrogen produced during N_2_ fixation is apparently recycled as demonstrated by the upregulation of the genes encoding a Ni/Fe-dependent hydrogenase. These hydrogenase genes were also upregulated under low oxygen conditions. Handling of nitrosative stress was shown by the expression of the nitric oxide reductase encoding genes *nor*B and *nor*C under all conditions tested, the upregulation of nitrite reductase *nir*K under oxygen limitation and of hydroxylamine oxidoreductase *hao* in the presence of ammonium. Unraveling the gene regulation of carbon and nitrogen metabolism helps to understand the underlying physiological adaptations of strain SolV in view of the harsh conditions of its natural ecosystem.

## Introduction

Methanotrophs are an unique group of microorganisms that can use methane (CH_4_) as sole carbon and energy source (Hanson and Hanson, 1996). Methanotrophs are found both in aerobic and anaerobic natural environments (Hanson and Hanson, 1996; Boetius et al., 2000; Raghoebarsing et al., 2006; Conrad, 2009). Aerobic methane-oxidizing bacteria are represented by members of the Alphaproteobacteria, the Gammaproteobacteria, the Verrucomicrobia, and the NC10 phylum (Hanson and Hanson, 1996; Op den Camp et al., 2009; Ettwig et al., 2010). “*Candidatus* *Methylomirabilis oxyfera*,” a representative of the latter phylum and growing anaerobically in the absence of oxygen, has the unique ability to produce intracellular oxygen through an alternative denitrification pathway (Ettwig et al., 2010).

During aerobic CH_4_ oxidation, energy is conserved during the oxidation of methanol, formaldehyde, and formate (Hanson and Hanson, 1996; Chistoserdova et al., 2009). In the oxidation of methanol, electrons are transferred to a membrane bound electron transport chain via a pyrroloquinoline quinone cofactor to cytochrome *c* and the bc1 complex by the enzyme methanol dehydrogenase. During formaldehyde and formate oxidation, NAD is reduced to NADH and transferred to NADH-oxidoreductase complex I (*nuo* genes). Electrons flow via the membrane protein complexes, Nuo, bc1, to the cytochrome *c* oxidases and produce a proton motive force that is converted to the cellular energy carrier ATP by the ATPase enzyme complex.

Verrucomicrobial methanotrophs were isolated from volcanic areas in Italy, New Zealand, and Russia (Dunfield et al., 2007; Pol et al., 2007; Islam et al., 2008) and, the genus name “*Methylacidiphilum*” was proposed since 16S rRNA gene sequences of the three independent isolates had 98–99% sequence identity (Op den Camp et al., 2009). Although environmental clone libraries from many ecosystems show a large abundance and biodiversity of Verrucomicrobia (Wagner and Horn, 2006), little is known about their *in situ* physiology. There are now several verrucomicrobial genome assemblies available (van Passel et al., 2011) including two of the verrucomicrobial methanotrophs (Hou et al., 2008; Khadem et al., 2012). The genome data of strains V4 and SolV showed some similarities but also major differences in the C1-utilization pathways compared to proteobacterial and NC10 methanotrophs. The functional significance of these differences can only be validated by a combination of physiological and expression studies.

Physiological studies of “*Ca. M. fumariolicum*” strain SolV have demonstrated that this microorganism was able to grow with ammonium, nitrate, or dinitrogen gas as nitrogen source (Pol et al., 2007; Khadem et al., 2010). ^13^C-labeling studies showed that strain SolV growing on CH_4_, fixed CO_2_ into biomass exclusively via the Calvin Benson Bassham (CBB) cycle (Khadem et al., 2011). Based on these results we expect that genes involved in nitrogen fixation are only expressed in the absence of ammonium/nitrate and genes involved in the CBB cycle are constitutively expressed. To evaluate this in more detail, analysis of the complete set of transcripts (the transcriptome) and their quantity present in cells grown under different condition is needed.

With the development of microarrays (Malone and Oliver, 2011) high-throughput quantification of the transcriptome became possible, improving the low throughput mRNA data from Northern blots or reverse-transcription PCR (RT-PCR) analysis. More recently, next generation sequencing has been shown to be a very powerful method to analyze the transcriptome of cells by what is known as RNA-Seq (Wang et al., 2009). Furthermore, this technique can detect transcripts without corresponding genomic sequences and can detect very low abundance transcripts (Croucher and Thomson, 2010; Malone and Oliver, 2011).

In this study we used RNA-Seq to analyze the genome wide transcriptome of “*Ca*. *M. fumariolicum*” SolV cells grown under different conditions at pH 2. Expression profiles of exponentially growing SolV batch cultures (at μ_max_) were compared to nitrogen fixing or oxygen limited chemostat cultures and used to unravel the gene and genome regulation of carbon and nitrogen metabolism which may reflect the underlying physiological adaptations of SolV.

## Materials and Methods

### Organism and medium composition for growth

“*Ca. Methylacidiphilum fumariolicum*” strain SolV used in this study was originally isolated from the Solfatara volcano, Campi Flegrei, near Naples, Italy (Pol et al., 2007).

Preparation and composition of the growth medium (pH 2) was described previously (Khadem et al., 2010). Mineral salts composition and concentration were changed for oxygen limited SolV chemostat cultures: 0.041 g l^−1^ MgCl_2_·6H_2_O was added (instead of 0.08 g l^−1^) and CaHPO_4_·2H_2_O was replaced by 0.138 g l^−1^ NaH_2_PO_4_.H_2_O to limit precipitation.

### Chemostat cultivation

Chemostat cultivation of strain SolV under nitrogen fixing condition at pH 2 was performed as described previously (Khadem et al., 2010). Growth yield and stoichiometry of CH_4_ conversion to CO_2_ of strain SolV were also determined for oxygen limited SolV chemostat cultures. The chemostat liquid volume was 300 ml and the system was operated at 55°C with stirring at 900 rpm with a stirrer bar. The chemostat was supplied with medium at a flow rate of 5.1 ml h^−1^, using a peristaltic pump. Culture liquid level was controlled by a peristaltic pump actuated by a level sensor. A gas mixture containing (v/v) 5.8% CH_4_, 2.3% O_2_, 0.4% N_2_, and 91.1% CO_2_ was supplied to the chemostat by mass flow controllers through a sterile filter and sparged into the medium just above the stirrer bar. Oxygen concentrations in the liquid were measured with a Clarke-type electrode.

After steady state was reached, CH_4_ and O_2_ consumption and CO_2_ production were determined by measuring the ingoing and outgoing gas flows and the gas concentrations. The outgoing gas passed through a sterile filter at a flow rate of 11.9 ml h^−1^, and contained (v/v) a mixture of approximately 4.8% CH_4_, 0.72% O_2_, 0.7% N_2_, and 92.7% CO_2_. The dissolved oxygen concentration (dO_2_) was below 0.03% oxygen saturation.

To determine biomass dry weight concentration, triplicate 5 ml samples from the culture suspension were filtered through pre-weighed 0.45 μm filters and dried to constant weight in a vacuum oven at 70°C. After steady state, both chemostats were sampled for mRNA isolation and Illumina sequencing.

### Batch cultivation

Cells of SolV grown at maximal growth rate (μ_max_), without any nitrogen, O_2_, and CH_4_ limitation were obtained in 1 liter serum bottles, containing 50 ml medium (with 4 mM ammonium, 2% fangaia soil extract, and at pH 2, Khadem et al., 2010) and sealed with red butyl rubber stoppers. Incubations were performed in duplicate and contained in (v/v) 10% CH_4_, 5% CO_2_, and 18% O_2_ at 55°C with shaking at 180 rpm. Exponentially growing cells were collected for mRNA isolation and Illumina sequencing.

### Gas and ammonium analyses

Gas samples (100 μl) were analyzed for methane (CH_4_), carbon dioxide (CO_2_), and oxygen (O_2_) on an Agilent series 6890 gas chromatograph (GC) equipped with Porapak Q and Molecular Sieve columns and a thermal conductivity detector as described before (Ettwig et al., 2008).

Ammonium concentrations were measured using the orthophthaldialdehyde (OPA) method (Taylor et al., 1974).

### Transcriptome analysis

The draft genome sequence of strain SolV (Khadem et al., 2012) was used as the template for the transcriptome analysis. Cells were harvested by centrifugation and 3.1 mg dry weight cells were used for isolation of mRNA, and subsequent synthesis of cDNA (328 ng) was done as described before (Ettwig et al., 2010). The cDNA was used for Illumina sequencing (RNA-Seq) as described before (Ettwig et al., 2010; Kartal et al., 2011). Expression analysis was performed with the RNA-Seq Analysis tool from the CLC Genomic Workbench software (version 4.0, CLC-Bio, Aarhus, Danmark) and values are expressed as RPKM (Reads Per Kilobase of exon model per Million mapped reads; Mortazavi et al., 2008).

## Results and Discussion

### Physiology of “*Ca*. *M. fumariolicum*” SolV growing with and without nitrogen source and under oxygen limitation

Prior to the expression studies the physiological properties of strain solV were examined in batch and chemostat continuous culture. These studies showed that strain SolV in batch culture had a maximum growth rate of 0.07 and 0.04 h^−1^, with ammonium or nitrate as nitrogen source, respectively (Table 1). In the absence of ammonium and nitrate “*Ca*. *M. fumariolicum*” SolV cells were able to fix atmospheric N_2_ only at headspace oxygen concentration below 1% (Khadem et al., 2010). The additional reduction steps of nitrate to ammonium could explain the observed increase in doubling time with nitrate compared to ammonium. The slower growth rate with N_2_ as nitrogen source was expected, since N_2_ fixation is an endergonic process, which needs about 16 mol ATP per mol N_2_ fixed (Dixon and Kahn, 2004). Based on the μ_max_ data obtained, strain SolV seems to prefer ammonium, which is also the most likely nitrogen source in its natural environment.

**Table 1:** **Description of batch and chemostat cultures of “*Ca. Methylacidiphilum fumariolicum*” SolV**.

Culture	Nitrogen source	Growth rate (h^−1^)	Doubling time (h)	Yield (g DW/mol CH_4_)	Limitation	O_2_ concentratio*n*^b^ (%)
Batch	Ammonium^a^	μ_max_ = 0.07	10	6.5	No	18
	Nitrate	μ_max_ = 0.04	17	n.d.	No	18
	N_2_	μ_max_ = 0.025	27	n.d.	Nitrogen	<1
Chemostat	N_2_^a^	μ = 0.017	40	3.5	Methane	0.5
	Ammonium^a^	μ = 0.017	40	4.9^c^	Oxygen	<0.03

^a^Cells used for transcriptome analysis. In all other Tables referred to as “Cells at μ_max_,” “N_2_ fixation,” and “O_2_ limitation.”

*^b^For batch cultures initial headspace oxygen concentrations are given, while for the chemostat cultures measured dissolved oxygen concentration (dO_2_) is expressed as % oxygen saturation (100% equals 800 μmol/l at 55°C)*.

*^c^Calculated from OD_600_ comparisons at steady state*.

Continuous cultivation of strain SolV cells in a chemostat at pH 2 under nitrogen fixing conditions, was performed at dissolved oxygen concentrations (dO_2_) equal to 0.5% oxygen saturation and without ammonium or nitrate (Table 1). Growth was limited by CH_4_ liquid-gas transfer in this chemostat culture (Khadem et al., 2010). The growth rate (0.017 h^−1^) is 68% of the μ_max_ (0.025 h^−1^) obtained in N_2_ fixing batch cultures.

For continuous cultivation of strain SolV under oxygen limitation and in the presence of excess methane and ammonium, the chemostat was supplied with medium at a dilution rate of 0.017 h^−1^ (Table 1). This resulted in a dO_2_ equal to 0.03% oxygen saturation (<0.24 μmol/l).

After a steady state was obtained in the chemostat, the stoichiometry of CH_4_ oxidation, and cell yield under N_2_ fixing and O_2_ limiting conditions were determined. Under O_2_ limitation the stoichiometry of CH_4_ oxidation was the same as reported, for excess ammonium and O_2_ (Pol et al., 2007). However, under N_2_ fixing conditions, a slightly higher consumption of O_2_ and production of CO_2_ was found (Khadem et al., 2010). This coincides with the lower cell yield of the nitrogen fixing chemostat culture of strain SolV (Table 1).

Three of the above described physiological conditions were selected for a genome wide transcriptome analysis (Table 1), e.g., exponentially growing cells (batch culture at μ_max_) and cells from nitrogen or oxygen limited chemostat cultures.

### Whole genome transcriptome analysis of “*Ca*. *M. fumariolicum*” SolV

**Figure A1:**
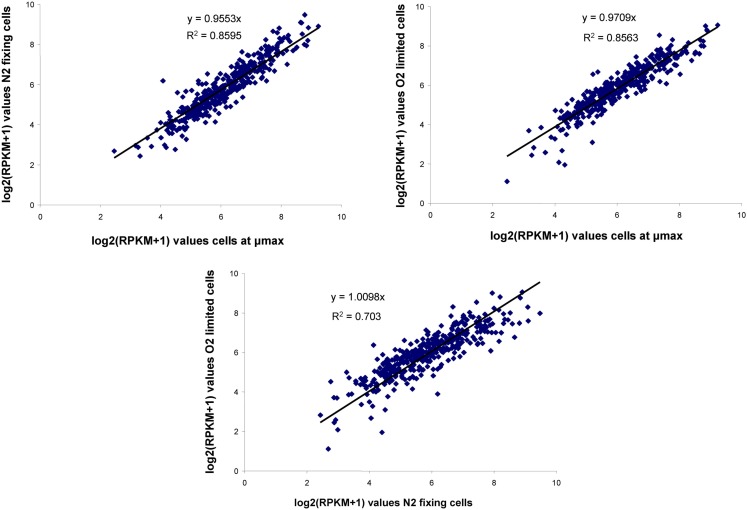
**Plots of expression levels (log2(RPKM + 1)) of 394 genes (in total 443 kbp) involved in energy generation, ribosomes, carbon fixation (CBB cycle), Cl metabolism (except for pmo), amino acid synthesis, cell wall synthesis, translation, transcription, DNA replication, and tRNA synthesis**.

The SolV transcriptome was characterized using RNA-Seq. RNA was prepared from the three different cell cultures (see above), converted to cDNA and sequenced. The Illumina Genome Analyzer reads (75 bp) were first mapped to the ribosomal RNA operon and mapped reads were discarded. The unmapped reads (3.5 × 10^6^, 3.2 × 10^6^, and 2.0 × 10^6^ reads for μ_max_, N_2_ fixing, and O_2_ limited cells, respectively) were mapped to the CDS, tRNA, and ncRNA sequences extracted from the genome sequence of strain SolV (Khadem et al., 2012). The total number of reads obtained and mapped for each sampled culture together with the calculated expression levels (RPKM) are provided File S1 in Supplementary Material (RNA-Seq_SolV.xls). We selected a set of 394 housekeeping genes (in total 443 kbp) involved in energy generation, in ribosome assembly, carbon fixation (CBB cycle), C1 metabolism (except for *pmo*), amino acid synthesis, cell wall synthesis, translation, transcription, DNA replication, and tRNA synthesis for the three conditions, to compare baseline expression levels (File S2 in Supplementary Material; Housekeeping genes.xls). For this gene set all ratios of expression between conditions were >0.5 and <2. The robustness of the transcriptome data were checked by the method of Chaudhuri et al. (2011) in which the expression levels (log2(RPKM + 1)) of the 394 gene set for the three conditions were plotted against each other. This resulted in correlation coefficients of 0.70, 0.86, and 0.86 (Figure A1 in Appendix), which are only slightly lower than those of technical replicates as reported by Chaudhuri et al. (2011).

In the following paragraphs, the differences in expression pattern under the various cultivation conditions with respect to energy, carbon, nitrogen, and hydrogen metabolism of strain SolV will be presented and discussed.

### Energy metabolism

**Figure 1:**
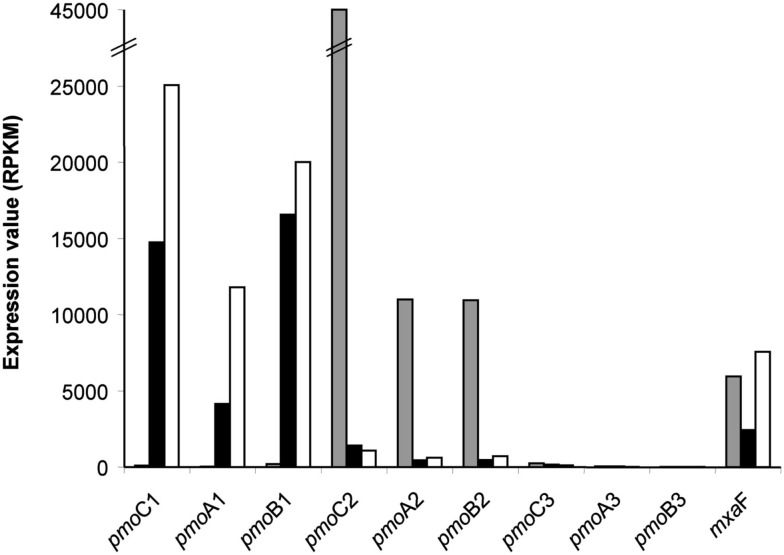
**Expression of the three *pmo*CAB operons, encoding the three subunits of the particulate methane monooxygenase (pMMO)**. Values from batch culture cells growing at μ_max_ ( 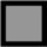 ), N_2_ fixing cells (■), and O_2_ limited cells (□) are expressed as RPKM (Mortazavi et al., 2008). Expression of *mxaF* (*xoxF*) encoding methanol dehydrogenase is shown for comparison.

Genes involved in CH_4_ oxidation pathway (Hanson and Hanson, 1996; Chistoserdova et al., 2009) and their RPKM values are presented in (Table 2). In the genome data of the verrucomicrobial methanotrophs no genes encoding for the soluble cytoplasmic form of the methane monooxygenase (sMMO) were found (Hou et al., 2008; Khadem et al., 2012). However, three *pmoCAB* operons, encoding for the three subunits of particulate membrane-associated form (pMMO) were predicted. Transcriptome analysis of “*Ca*. *M. fumariolicum*” SolV showed differential expression of two of the three different operons. One of the *pmoCAB* operons (*pmoCAB*2) was highly expressed (RPKM values 10.9 × 10^3^ to 45 × 10^3^, Figure 1) in cells growing at μ_max_ with excess ammonium and oxygen (initial headspace concentration of 18%). The other two *pmoCAB* operons were hardly expressed under these culture conditions (RPKM 21–253). The cells from CH_4_ limited N_2_ fixing chemostat culture and the O_2_ limited chemostat culture with dO_2_ of 0.5 and 0.03% oxygen saturation, respectively, showed a remarkable different expression pattern of the *pmoCAB* operons. Under these conditions the *pmoCAB*1 operon was highly expressed (RPKM values 4.1 × 10^3^ to 25 × 10^3^) while expression of the *pmoCAB*2 operon was down regulated 40 times compared to the batch culture. The *pmoCAB*3 operon was hardly expressed in cells from the two chemostat cultures, expression values being identical to that of the cells at μ_max_. Although other factors like growth rate, cell density, etc., could have an effect, the results point to a regulation of the *pmoCAB*1/*pmoCAB*2 genes by the oxygen concentration. Since the *pmoCAB*3 operon was not expressed under the conditions tested, other growth conditions have to be tested to elucidate the regulation and function of this pMMO. In a recent study, qPCR was used to investigate expression of the four *pmoA* genes of “*Ca. M. kamchatkense*” Kam1 (Erikstad et al., 2012). The *pmoA*2 gene was 35-fold stronger expressed than the other copies. Suboptimal temperature and pH conditions did not change this pattern. Other limitations were not tested. Grow on methanol resulted in a 10-fold decreased expression of *pmoA*2.

**Table 2:** **Transcription of genes involved in oxidation of CH_4_ in “*Ca. M. fumariolicum*” strain SolV**.

Enzyme	Gene name	GenBank identifier	Expression level (RPKM)		
			Cells at μ_max_	N_2_ fixing cells	O_2_ limited cells
Methane monooxygenase_1	*pmoC1*	Mfum_790003	90	14764	25054
	*pmoA1*	Mfum_790002	37	4148	11804
	*pmoB1*	Mfum_790001	181	16550	20004
Methane monooxygenase_2	*pmoC2*	Mfum_780001	45059	1405	1087
	*pmoA2*	Mfum_770004	10994	454	598
	*pmoB2*	Mfum_770003	10930	467	712
Methane monooxygenase_3	*pmoC3*	Mfum_480006	253	148	101
	*pmoA3*	Mfum_480005	45	44	24
	*pmoB3*	Mfum_480007	21	17	15
Methanol dehydrogenase	*mxaF/xoxF*	Mfum_190005	5945	2434	7554
Periplasmic binding protein	*mxaJ*	Mfum_190004	941	1548	629
Cytochrome *c* family protein	*mxaG*	Mfum_190003	760	513	522
Coenzyme PQQ synthesis proteins	*pqqB*	Mfum_80011	483	288	677
	*pqqC*	Mfum_80010	589	645	1243
	*pqqD*	Mfum_710019	32	26	55
	*pqqD*	Mfum_80009	108	118	195
	*pqqE*	Mfum_80008	525	1078	766
	*pqqF*	Mfum_690050	527	339	737
NADPH:quinone reductase	*qor1*	Mfum_270035	330	236	1092
	*qor2*	Mfum_300032	608	690	509
	*qor3*	Mfum_820025	117	77	128
Zn-dependent alcohol dehydrogenase	*adhP1*	Mfum_310051	216	397	218
	*adhP2*	Mfum_680019	165	96	196
Aldehyde dehydrogenase	*dhaS1*	Mfum_940074	58	68	73
	*dhaS2*	Mfum_840001	1345	1008	1337
7,8-Dihydropteroate synthase	*folP1*	Mfum_690066	199	162	236
	*folP2*	Mfum_940066	163	236	133
Formate-tetrahydrofolate ligase	*fhs*	Mfum_300027	286	279	371
Bifunctional protein	*folD*	Mfumv1_210029	162	89	112
GTP cyclohydrolase	*folE*	Mfumv1_990006	993	501	1135
Formate dehydrogenase	*fdsA*	Mfumv1_80015	987	645	763
	*fdsB*	Mfumv1_80014	675	905	520
	*fdsC*	Mfumv1_80013	298	75	182
	*fdsD*	Mfumv1_80016	313	260	62
Formate dehydrogenase	*fdh*	Mfumv1_50001	233	186	257
Methylamine dehydrogenase	*mauA*	Mfumv1_700106	45	24	101
	*mauB*	Mfumv1_700109	220	199	168

*The mRNA expression is expressed as RPKM according to Mortazavi et al. (2008). Changes in expression in the chemostat cultures (N_2_ fixing cells or O_2_ limited cells) compared to batch culture cells growing at μ_max_ are indicated by shading: up regulation >2 times (dark gray), down regulation <0.5 (light gray)*.

Also some proteobacterial methanotrophs are known to contain multiple copies of *pmo* operons (Semrau et al., 1995; Murrell et al., 2000). Within sequenced genomes of gammaproteobacterial methanotrophs two nearly sequence-identical copies of *pmoCAB1* were found. It is thought that sequence-identical copies have arisen through gene duplications and insertion. Mutation studies in *Methylococcus* *capsulatus* Bath have demonstrated that both pMMO’s were required for growth (Stolyar et al., 1999). More sequence-divergent copies (*pmo*CAB2) were shown to be widely distributed in alphaproteobacterial methanotrophs (Yimga et al., 2003). Recently it was found that some genera of gammaproteobacterial methanotrophs also posses a sequence-divergent particulate methane monooxygenase, depicted pXMO (Tavormina et al., 2011). Unlike the CAB gene order of the *pmo* operon the *pxm* operon shows an ABC gene order. The presence of sequence-divergent copies suggests alternative physiological function under different environmental conditions. *Methylocystis* sp. strain SC2 was shown to possess two pMMO isozymes, encoded by *pmoCAB*1 and *pmoCAB*2 operons. The pmo*CAB*1 operon was expressed by strain SC2 at mixing ratios >600 ppmv CH_4_, while growth and concomitant oxidation of methane at concentrations <600–700 ppmv was due to the expression of *pmoCAB*2 (Baani and Liesack, 2008). In this case the methane concentration seems to control the up- and down regulation of the different pMMOs.

The second step in CH_4_ oxidation pathway is the conversion of methanol to formaldehyde by methanol dehydrogenase. Methanol dehydrogenase activity in strain SolV could be demonstrated but the gene cluster encoding this activity seems to be rather different compared to proteobacterial methanotrophs. The *mxaFJGIRSACKLDEH* cluster encoding the methanol dehydrogenase (*mxaFI*), a cytochrome (*mxaG*), a solute binding protein (*mxaJ*), and accessory proteins (Chistoserdova et al., 2003; Ward et al., 2004; Chen et al., 2010) was absent in the verrucomicrobial methanotrophs (Hou et al., 2008; Khadem et al., 2012) and found to be replaced by a *mxaFJG* operon. In addition the gene cluster *pqqABCDEF* encoding proteins involved in biosynthesis of the methanol dehydrogenase cofactor pyrroloquinoline quinone was present. The expression of these genes did not vary much under the conditions tested (Table 2; Figure 1).

Formaldehyde, the product from the methanol dehydrogenase, is a key intermediate in methanotrophs. It may be oxidized for energy and detoxification, or fixed into cell carbon via the ribulose monophosphate pathway (RuMP) or serine cycle (see below, Hanson and Hanson, 1996; Chistoserdova et al., 2009). The canonical formaldehyde oxidation pathway requires folate as a cofactor for C_1_ transfer and formate dehydrogenase complexes (see below). The classical gene *folA* involved in the last step of folate-biosynthesis (encoding dihydrofolate reductase, FolA) is absent in “*Ca. Methylacidiphilum*” strains V4 and SolV. Hou et al. (2008) suggested that the role of this enzyme could be taken over by an alternative dihydropteroate synthase (FolP). The gene encoding for this enzyme was also present in strain SolV and was constitutively expressed at RPKM values of 133–236. The presence of the *folD* gene in the “*Ca. Methylacidiphilum*” strains (V4 and SolV) and expression data of this gene in strain SolV (Table 2), indeed suggest that conversion of formaldehyde is tetrahydrofolate-dependent. The “archaeal” tetrahydromethanopterin cofactor-based pathway for C1 transfer found in other methylotrophs is not present in the genomes of the “*Ca. Methylacidiphilum*” strains (Hou et al., 2008; Khadem et al., 2012).

Formaldehyde can also directly be oxidized by a formaldehyde dehydrogenase. The genome data of stain SolV reveal several candidates for formaldehyde oxidation: a NADPH:quinone reductase (or related Zn-dependent oxidoreductases), Zn-dependent alcohol dehydrogenases or the NAD-dependent aldehyde dehydrogenases. The genes encoding for these enzymes were expressed under all conditions tested (Table 2). A role for these enzymes should be further supported by enzyme purification and characterization studies. Genes encoding for soluble and membrane bound NAD-dependent formate dehydrogenases were also predicted from the draft genome of strain SolV and there expression levels were not significantly different the same under all experimental conditions (Table 2). This enzyme performs the last step of CH_4_ oxidation, converting formate into CO_2_.

### Carbon metabolism (carbon fixation)

The genome data of the verrucomicrobial methanotrophs (Hou et al., 2008; Khadem et al., 2012) showed differences in carbon assimilation compared to proteobacterial methanotrophs (Chistoserdova et al., 2009). Analyses of the draft genome of “*Ca*. *M. fumariolicum*” strain SolV revealed that the key genes needed for an operational RuMP pathway, hexulose-6-P synthase and hexulose-6-P isomerase were absent. In addition, the crucial genes encoding key enzymes of the serine pathway, malyl coenzyme A lyase, and glycerate kinase, were not found (Khadem et al., 2011). However, all genes required for an active CBB cycle could be identified in the SolV genome. These genes were highly expressed in both chemostat cultures (Table A1 in Appendix), to levels identical to those of cells in batch cultures growing at μ_max_ (Khadem et al., 2011). The constitutive expression in all cell cultures was expected, assuming biomass carbon in strain SolV growing on methane can only be derived from fixation of CO_2_ via the CBB cycle (Khadem et al., 2011). Our transcriptome data of the chemostat cultures and batch cultures showed low expression of the *cbbR* gene, encoding a possible RuBisCO operon transcriptional regulator. The *cbbR* gene product is a LysR-type transcriptional regulator and the key activator protein of *cbb* operons in facultative autotrophs (Bowien and Kusian, 2002). As an autotroph, strain SolV may not need much regulation of the CBB cycle genes. For strain V4 a coupling of this *cbbR* gene to nitrate reduction and transport was suggested (Hou et al., 2008).

Although, the genes encoding for the ribulose-1,5-bisphosphate carboxylase/oxygenase (RuBisCO), the key enzyme of the CBB cycle was found in the genome of some proteobacterial methanotrophs like *M. capsulatus* Bath (Ward et al., 2004) and *Methylocella silvestris* BL2 (Chen et al., 2010) and the non-proteobacterial methanotroph “*Candidatus* Methylomirabilis oxyfera” (Ettwig et al., 2010), autotrophic growth in liquid cultures has not been reported for these methanotrophs yet.

### Carbohydrate metabolism

The presence and transcription of genes involved in the pentose phosphate pathway suggested the possibility of gluconeogenesis in strain SolV (Table A1 in Appendix). In *M*. *capsulatus* Bath, gluconeogenesis was suggested as follows: a putative phosphoketolase, condenses pyruvate, and glyceraldehyde-3-phosphate into xylulose-5-phosphate, which in turn is fed into the ribulose-5-phosphate pool for formation of glucose-6-phosphate through the pentose phosphate pathway (Ward et al., 2004). Since a putative phosphoketolase is also present and expressed in strain SolV, gluconeogenesis might take place in the same way. Another possibility for the production of glucose-6-phosphate from glyceraldehyde-3-phosphate would be the consecutive action of triose-P-isomerase, fructose-1,6-bisphosphate aldolase, fructose-1,6-bisphosphate phosphatase, and glucose-6-P isomerase. All genes encoding these enzymes are expressed under the growth conditions tested (Table A1 in Appendix).

In many gammaproteobacterial methanotrophs, the tricarboxylic acid (TCA) cycle is believed to be incomplete, because they lack the α-ketoglutarate dehydrogenase activity (Hanson and Hanson, 1996). However in the *M*. *capsulatus* genome homologs of this enzyme were indentified, suggesting that the TCA cycle might operate in this microorganism (Ward et al., 2004). Alphaproteobacterial methanotrophs are known to have a complete TCA cycle (Hanson and Hanson, 1996; Dedysh et al., 2002; Chen et al., 2010). The genes encoding for the TCA cycle enzymes were predicted from the genomes of strains V4 and SolV (Hou et al., 2008; Khadem et al., 2012). Our transcriptome analysis showed that these genes were expressed under the conditions applied, with slightly lower expression levels under N_2_ fixing conditions (Table A2 in Appendix). The presence of an operational TCA cycle in strain SolV suggests that growth on two carbon compounds like acetate should be possible. The presence and transcription of a gene encoding acetyl-coenzyme A synthetase (*acs*), allows acetate to be activated and fed into the TCA cycle (Table A2 in Appendix). Three alphaproteobacterial genera *Methylocella*, *Methylocapsa*, and *Methylocystis*, which were shown to be able to grow or survive on acetate, also posses a TCA cycle (Dedysh et al., 2005; Dunfield et al., 2010; Belova et al., 2011; Semrau et al., 2011).

### Potential carbon and energy storage

Many bacteria start to accumulate reserve polymers when enough supply of suitable carbon is available, but nitrogen is limited (Wanner and Egli, 1990). This phenomenon is also known for methanotrophs (Pieja et al., 2011). Recently it was shown that type II methanotrophs contained the gene *pha*C, which encodes for the poly-3-hydroxybutyrate (PHB) synthase enable them to produce PHB (Pieja et al., 2011). At least three genes (*phaC*, *phaA*, *phaB*) were considered to be crucial for PHB synthesis. These genes are absent in type I methanotrophs and in the “*Ca. Methylacidiphilum*” strains (Hou et al., 2008; Khadem et al., 2012). However, genes encoding for glycogen synthesis, degradation, and transport (glycogen synthase, glycogen debranching enzyme, and ADP-glucose pyrophosphorylase) were predicted based on the draft genome of strain SolV. These genes were expressed under all conditions tested (Table A3 in Appendix). This supports the ability of carbon storage by strain SolV, but further physiological studies with cells growing under excess of carbon and nitrogen limitation are needed. Thus far literature on glycogen synthesis in methanotrophs is sparse, but several of the publicly available genomes of proteobacterial methanotrophs contain glycogen synthesis genes (*M. capsulatus* str. Bath; *Methylomonas methanica* MC09; *Methylomicrobium alcaliphilum*; *Methylocystis* sp. ATCC 49242; http://www.ncbi.nlm.nih.gov/genomes).

The presence and constitutive expression of genes involved in phosphate transport, polyphosphate synthesis, and utilization (ABC-type phosphate transport system, polyphosphate kinase, adenylate kinase, and exopolyphosphatase; Table A3 in Appendix) suggest that strain SolV is able to store polyphosphate as energy and phosphorus reserve.

### Nitrogen metabolism: Ammonium, nitrate, and amino acid metabolism

Based on the genome and supported by the transcriptome data the main route for ammonium assimilation in “*Ca*. *M. fumariolicum*” occurs via glutamine synthase (*glnA*)/glutamate synthase (*gltB*) and/or the alanine and glutamate dehydrogenases (*ald*, *gdh*). Expression values of *ald* and *gdh* were about three- to fivefold lower compared to *gln*A and *glt*B under the conditions tested (Table 3). Also the genes encoding the glutamine-hydrolyzing carbamoyl-phosphate synthase (*carA* and *carB*) were constitutively expressed. This enzyme coverts glutamine and carbon dioxide into glutamate and carbamoyl-phosphate. The latter substrate can be fed into the urea cycle. Except for the gene encoding arginase all other genes (*argDHFG*) encoding enzymes of the urea cycle were present and constitutively expressed. The most likely function of this partial cycle will be arginine synthesis. For strain V4 it was suggested that the ornithine needed can be supplied by 4-aminobutyrate aminotransferase through a part of the TCA cycle and glutamate synthesis (Hou et al., 2008). In strain SolV, the gene encoding 4-aminobutyrate aminotransferase is also present and expressed. Other methylotrophs possess neither arginase nor ArgD (Hou et al., 2008).

**Table 3:** **Transcription of genes involved in nitrogen metabolism in “*Ca. M. fumariolicum*” strain SolV**.

Enzyme	Gene name	GenBank identifier	Expression level (RPKM)		
			Cells at μ_max_	N_2_ fixing cells	O_2_ limited cells
Glutamine synthetase	*glnA*	Mfum_90015	1542	1052	725
Glutamine synthetase regulatory protein PII	*glnB*	Mfum_90016	1039	1701	914
UTP:GlnB (Protein PII) uridylyltransferase	*glnD*	Mfum_230007	169	111	145
Nitrogen regulatory protein PII	*glnK*	Mfum_140026	166	60	203
Alanine dehydrogenase	*ald*	Mfum_290047	248	279	256
Glutamate dehydrogenase	*gdhA*	Mfum_810044	436	182	500
Glutamate synthase alpha chain	*gltB*	Mfum_940063	1343	1360	1355
Glutamate synthase beta chain	*gltD*	Mfum_270076	133	114	125
Ornithine/acetylornithine aminotransferase	*argD1*	Mfum_190040	736	736	410
	*argD2*	Mfum_1010035	383	421	410
Argininosuccinate lyase	*argH*	Mfum_970020	226	186	107
Ornithine carbamoyltransferase	*argF*	Mfum_1010036	286	267	375
Argininosuccinate synthase	*argG*	Mfum_270005	725	527	678
Carbamoyl-phosphate synthase small chain	*carA*	Mfum_270024	450	340	479
Carbamoyl-phosphate synthase large chain	*carB*	Mfum_700048	395	267	504
Ammonium transporter	*amtB*	Mfum_430001_160001^a^	343	1143	420
Nitrate ABC transporter, nitrate-binding protein	*tauA*	Mfum_140012	28	117	33
Nitrate transporter	*nasA*	Mfum_140017	22	26	16
Assimilatory nitrate reductase large subunit	*bisC*	Mfum_140014	13	14	8
Assimilatory nitrite reductase	*nirB*	Mfum_140015	14	40	13
Ferredoxin subunit of nitrite reductase	*nirD*	Mfum_140016	32	18	16
Signal transduction histidine kinase with PAS domain	*ntrB*	Mfum_920004	283	294	250
Signal transduction response regulator, NtrC family	*ntrC1*	Mfum_110018	90	76	67
	*ntrC2*	Mfum_170043	116	110	111
	*ntrC3*	Mfum_260013	623	361	525
	*ntrC4*	Mfum_920003	225	154	216
Hydroxylamine oxidoreductase	*haoA*	Mfum_970027	357	124	568
Nitric oxide reductase B subunit	*norB*	Mfum_970100	261	120	342
Nitric oxide reductase subunit C	*norC*	Mfum_970099	192	93	139
Nitrite reductase (NO-forming)	*nirK*	Mfum_270071	72	63	200
DNA-binding response regulator, NarL family	*mxaB*	Mfum_1030004	232	74	142
DNA-binding response regulator, LuxR family	*citB1*	Mfum_790006	1760	5611	4679
DNA-binding response regulator, LuxR family	*citB2*	Mfum_580001	394	200	239

*The mRNA expression is expressed as RPKM according to Mortazavi et al. (2008). Changes in expression in the chemostat cultures (N_2_ fixing cells or O_2_ limited cells) compared to batch culture cells growing at μ_max_ are indicated by shading: up regulation >2 times (dark gray), down regulation <0.5 (light gray). ^a^Mfum_430001 and Mfum_160001 encode the N- and C-terminal part, respectively*.

The genes encoding nitrate/nitrite transporters and the assimilatory nitrite and nitrate reductases showed very low expression levels (8–117), probably due to the absence of nitrate in the growth media used. The ammonium transporter gene (*amtB* type) is three- to fourfold upregulated in N_2_ fixing cells, which reflects increased ammonium scavenging under nitrogen limited conditions.

### Nitrogen fixation

The genomes of strain SolV and strain V4 show a complete set of genes necessary for N_2_ fixation (Hou et al., 2008; Khadem et al., 2012). Most of these genes and their organization in putative operons resemble those of *M. capsulatus* Bath (Ward et al., 2004), a gammaproteobacterial methanotroph that has been shown to fix atmospheric N_2_ (Oakley and Murrell, 1991). N_2_ fixation is widely distributed among methanotrophs as shown by the presence of both *nifH* gene fragments and acetylene reduction activity in a variety of alpha- and gammaproteobacterial methanotroph strains (Auman et al., 2001). Also the deep-sea anaerobic methane-oxidizing Archaea were shown to fix N_2_ and share the products with their sulfate-reducing bacterial symbionts (Dekas et al., 2009).

Gene expression data of strain SolV showed that all the genes involved in nitrogen fixation were upregulated only in absence of ammonium and nitrate indicating the effect of nitrogen availability on the expression of these genes (Table 4). The genes encoding for the nitrogenase (*nifH*, *nifD*, *nifK*) were 100- to 325-fold upregulated, while the gene involved in regulation (nif*A*) and the Fe/Mo cofactor biosynthesis genes showed 30- to 235-fold increased expression levels. Our previous physiological studies already confirmed that nitrogenase was active in N_2_ fixing chemostat cultures (Khadem et al., 2010).

**Table 4:** **Transcription of genes involved in nitrogen fixation in “*Ca*. *M. fumariolicum*” strain SolV**.

Enzyme	Gene name	GenBank identifier	Expression level (RPKM)		
			Cells at μ_max_	N_2_ fixing cells	O_2_ limited cells
Nitrogenase iron protein	*nifH*	Mfum_690040	69	22651	80
Nitrogenase molybdenum-iron protein alpha chain	*nifD*	Mfum_690039	87	14359	65
Nitrogenase molybdenum-iron protein beta chain	*nifK*	Mfum_690038	35	3348	34
Nitrogenase Mo/Fe cofactor biosynthesis protein NifE	*nifE*	Mfum_690037	77	5370	67
Nitrogenase Mo/Fe cofactor biosynthesis protein NifN	*nifN*	Mfum_690036	64	3677	52
Protein NifX	*nifX*	Mfum_690035	100	2831	26
Nif-specific regulatory protein	*nifA*	Mfum_690018	123	1080	109
Nitrogenase Mo/Fe cofactor biosynthesis protein NifB	*nifB*	Mfum_690029	19	3316	14
Pyruvate-flavodoxin oxidoreductase	*nifJ*	Mfum_940083	149	133	185
NifQ family protein	*nifQ*	Mfum_690020	65	270	22
Cysteine desulfurase	*nifS1*	Mfum_690022	93	801	87
	*nifS2*	Mfum_90010	159	133	116
	*nifS3*	Mfum_190023	681	491	486
	*nifS4*	Mfum_970062	162	275	145
	*nifS5*	Mfum_310028	226	151	264
NifU-like protein involved in FeS cluster formation	*nifU*	Mfum_310029	54	16	79
Nitrogenase-stabilizing/protective protein NifW	*nifW*	Mfum_690011	62	2260	43
NifZ domain protein	*nifZ*	Mfum_690023	158	1373	50
Electron transfer flavoprotein beta chain	*fixA*	Mfum_690010	60	3311	73
Electron transfer flavoprotein alpha chain	*fixB*	Mfum_690009	81	3043	69
Flavoprotein-ubiquinone oxidoreductase	*fixC*	Mfum_690008	101	3632	100
Ferredoxin-like protein	*fixX*	Mfum_690007	126	2917	135
Nitrogen fixation protein FixU	*fixU*	Mfum_690015	164	2265	91
FeS cluster assembly scaffold protein, HesB/SufA family	*sufA1*	Mfum_690026	40	2301	22
	*sufA2*	Mfum_810040	149	43	71
	*sufA3*	Mfum_1020116	214	143	183
FeS cluster assembly protein SufB	*sufB*	Mfum_970056	1099	2409	622
FeS cluster assembly protein SufD	*sufD*	Mfum_970057	663	2169	279
FeS cluster assembly protein SufE family	*sufE*	Mfum_110022	375	209	219
FeS4 cluster protein with leucine rich repeats		Mfum_690024	88	1884	61
Ferredoxin-like protein in *nif* region	*frxA*	Mfum_690027	112	7892	46
Uptake hydrogenase large subunit	*hupB*	Mfum_50004	92	372	1840
Uptake hydrogenase small subunit	*hupS*	Mfum_50003	186	997	2955
Nickel/iron-hydrogenase I, small subunit	*hyaA*	Mfum_870019	2380	4157	4280
Nickel/iron-hydrogenase I, large subunit	*hyaB*	Mfum_870018	2566	1879	3595
Ni,Fe-hydrogenase I cytochrome *b* subunit	*hyaC*	Mfum_50005	122	593	984
[NiFe] hydrogenase Ni incorporation protein HypA	*hypA*	Mfum_730023	119	110	95
[NiFe] hydrogenase Ni incorporation-associated protein HypB	*hypB*	Mfum_870009	421	335	692
[NiFe] hydrogenase metallocenter assembly protein HypC	*hypC*	Mfum_870006	830	600	321
[NiFe] hydrogenase expression/formation protein HypD	*hypD*	Mfum_870005	1159	893	1246
[NiFe] hydrogenase metallocenter assembly protein HypE	*hypE*	Mfum_870004	424	476	759
[NiFe] hydrogenase metallocenter assembly protein HypF	*hypF*	Mfum_870007	92	81	167
Hydrogenase expression protein HupH	*hupH*	Mfum_50006	160	631	879
Hydrogenase expression/formation protein HoxQ	*hoxQ*	Mfum_730017	177	169	136
Hydrogenase maturation protease	*hycI*	Mfum_730022	69	44	89

*The mRNA expression is expressed as RPKM according to Mortazavi et al. (2008). Changes in expression in the chemostat cultures (N_2_ fixing cells or O_2_ limited cells) compared to batch culture cells growing at μ_max_ are indicated by shading: up regulation >2 times (dark gray), down regulation <0.5 (light gray)*.

Growth on atmospheric nitrogen in the chemostat was only observed when the dO_2_ was below 0.5% oxygen saturation. Our previous batch incubations in the presence of ammonium and 0.5% O_2_ saturation resulted in doubling time of 10 h (Khadem et al., 2010). This indicates that in N_2_ fixing chemostat cultures, this low oxygen was not growth limiting. Maintaining a low oxygen concentration in both batch and chemostat is required for an active nitrogenase, since this enzyme is irreversibly damaged by O_2_ (Robson and Postgate, 1980). Low oxygen requirement for N_2_ fixation was also demonstrated for other proteobacterial methanotrophs (Murrell and Dalton, 1983; Takeda, 1988; Dedysh et al., 2004). The effect of high oxygen concentration on the expression of genes encoding N_2_ fixing enzymes, in absence of ammonium/nitrate still needs to be addressed in strain SolV.

Methanotrophic hydrogenases are considered to have a role in N_2_ fixation or CH_4_ oxidation. The role of hydrogenase as a source of reducing power for CH_4_ oxidation was demonstrated in *M. capsulatus* Bath (Hanczar et al., 2002). Hydrogen uptake and evolution activities during N_2_ fixation were reported for *Methylosinus trichosporium* (De Bont and Mulder, 1976) and *Methylocystis* T-1 (Takeda, 1988), respectively. However, knock out studies of *ΔhupSL* encoding for the large and small subunit of the Ni/Fe-dependent hydrogenase in *M*. *capsulatus* Bath, did not show differences in viability under nitrogen fixing and non-nitrogen fixing condition in comparison to the wild strain (Csaki et al., 2001). Based on these results, the authors suggested that the hydrogenase is probably regulated by oxygen availability rather than by the hydrogen generated by the nitrogenase enzyme complex. Our expression data also show an increased expression under both nitrogen fixing and oxygen limited conditions (Table 4). Since under oxygen limitation the nitrogen fixing genes were not expressed, while the hydrogenase encoding genes were expressed to even higher levels, oxygen seems to be the regulatory factor for the latter set of genes.

The PII signal transduction proteins (encoded by *glnB* and *glnK*) are used to transduce the nitrogen status of the cell to the NtrB–NtrC two-component regulatory system and the σ^54^-dependent *amtB* promoters to tune *nif* gene transcription (for a detailed overview see Dixon and Kahn, 2004). The *glnB* gene of strain SolV was highly expressed under all conditions and slightly upregulated (1.5-fold) under N_2_ fixing conditions. Expression of *glnK* was overall about fivefold lower and threefold downregulated under nitrogen fixing conditions. In addition genes encoding for uridylyltransferase (*glnD*), NtrB, and NtrC showed expression levels under three conditions tested which did not significantly differ (Table 3). This suggests that in strain SolV the PII proteins are involved in sensing and regulating the status of fixed nitrogen in the cell. Transcription of the *nif* genes is regulated by *nifA* and *nifL* genes (Dixon and Kahn, 2004). The expression of *nifA* is regulated by oxygen and/or fixed nitrogen and *nifL* gene is involved in oxygen sensing. We could not identify a *nifL* gene in the genome of strain SolV (Khadem et al., 2012). However, *nifA* is present and was 30-fold upregulated under N_2_ fixing conditions.

Nitrogenase is believed to be sensitive for reactive oxygen species (ROS), and during nitrogen fixation the level of ROS is reduced by up regulation of ROS-detoxifying genes. In *Gluconacetobacter diazotrophicus* upregulation of these genes was observed during nitrogen fixation (Alquéres et al., 2010). Although, in the genome of strain SolV two *sodA* genes encoding for superoxide dismutases can be identified, they both are highly expressed under all conditions tested (RPKM values: Mfum_810007, 797 ± 163; Mfum_980001, 961 ± 669), but expression seems to be 1.5- to 3-fold lower under N_2_ fixing and O_2_ limited conditions.

### Nitrosative stress

The pMMO enzyme involved in the first step of CH_4_ oxidation in methanotrophs, also oxidizes ammonium which results in the formation of the intermediate hydroxylamine (NH_2_OH; Hanson and Hanson, 1996; Nyerges and Stein, 2009 and reference therein; Stein and Klotz, 2011). Ammonia-oxidizers can relay electrons from hydroxylamine oxidation to the quinone pool to drive energy production and cellular growth (Klotz and Stein, 2008), but methanotrophs lack this relay and can not produce energy from this oxidation. Since hydroxylamine is a highly toxic intermediate, methanotrophs rely on mechanisms to quickly remove it. In their natural habitat the “*Ca*. *M. fumariolicum*” cells are confronted with varying nitrogen levels (1–28 mM, Khadem et al., 2010) which means that the cells have to balance assimilation and tolerance to reactive-N. Detoxification can be achieved by conversion of hydroxylamine back to ammonium or to nitrite through the use of a hydroxylamine reductase enzyme (HAO). The nitrite in turn can be converted to N_2_O via NO by putative denitrifying enzymes (nitrite reductase and NO-reductase, Campbell et al., 2011). Genes involved in these conversions may include *hao*, *cytL*, *cytS*, *nirBD*, *nirS* or *nirK*, and *norCB*. The genes *hao*, *norCB* were shown to be present in the genomes of the verrucomicrobial methanotrophs (Hou et al., 2008; Khadem et al., 2012), while a *nirK* homolog was only found in strain SolV. The gene inventory in methanotrophic bacteria for handling hydroxylamine or other toxic nitrosating intermediates and for those encoding putative denitrifying enzymes is diverse and unpredictable by phylotype or taxon (Stein and Klotz, 2011).

In our study we found that although expressed under all conditions tested, expression of *hao* and *norBC* were 1.5- to 4.5-fold lower under nitrogen fixing conditions (Table 3), which makes sense in view of the expected lower ammonium levels in the cells. However, for *nirK* expression was low (RPKM = 63–72) except for the cells grown under oxygen limitation (RPKM = 200).

## Conclusion

In this study we analyzed the genome wide changes in expression during three different growth conditions which helped very much to understand the physiology of “*Ca*. *M. fumariolicum*” strain SolV. The analysis indicated that the two of the three *pmo*CAB operons are probably regulated by oxygen, although the effect of other factors like growth rate, cell density can not be excluded. Further, the hydrogen produced during N_2_ fixation can be recycled, and that nitrosative stress is counter acted. the results point to a regulation of the *pmoCAB*1/*pmoCAB*2 genes by the oxygen concentration.

The obtained information will be a guide to design future physiological and biochemical studies.

## Conflict of Interest Statement

The authors declare that the research was conducted in the absence of any commercial or financial relationships that could be construed as a potential conflict of interest.

## Supplementary Material

The Supplementary Material for this article can be found online at http://www.frontiersin.org/Evolutionary_and_Genomic_Microbiology/10.3389/fmicb.2012.00266/abstract

File S1 RNA-Seq analysis of “*Ca*. *Methylacidiphilum fumariolicum*” SolV grown under different conditions.

File S2 Housekeeping genes to test robustness of transcriptome data.

## Appendix

**Table A1:** **Transcription of genes involved in carbon fixation and the pentose phosphate pathway in “*Ca. M. fumariolicu**m*” strain SolV**.

Enzyme	Gene name	GenBank identifier	Expression level (RPKM)		
			Cells at μ_max_	N_2_ fixing cells	O_2_ limited cells
6-Phosphogluconolactonase	*nagB*	Mfum_380003	166	70	285
Glucose-6-phosphate isomerase	*pgi*	Mfum_180006	218	156	112
Ribose-5-phosphate-3-epimerase	*rpe*	Mfum_830014	238	227	335
Transaldolase	*mipB*	Mfum_70005	279	301	256
Phosphoketolase	*xfp*	Mfum_200044	326	253	302
Transaldolase	*mipB*	Mfum_120006	380	205	187
Glucose-6-phosphate 1-dehydrogenase	*zwf*	Mfum_380002	569	478	469
Triosephosphate isomerase	*tpiA*	Mfum_170033	587	428	522
Fructose-1,6-bisphosphatase II	*glpX*	Mfum_1080012	591	670	750
6-Phosphogluconate dehydrogenase	*gnd*	Mfum_1020039	610	334	411
Glucose-6-phosphate 1-dehydrogenase	*zwf*	Mfum_1020106	686	302	794
Ribose 5-phosphate isomerase B	*rpiB*	Mfum_1020038	752	335	1250
Phosphoribulokinase, chromosomal	*cfxP*	Mfum_280006	829	682	662
Phosphoglycerate kinase	*pgk*	Mfum_170032	891	1066	671
Fructose-1,6-bisphosphatase class 1	*fbp*	Mfum_280007	1174	974	1078
Phosphoribulokinase	*udk*	Mfum_70009	1798	938	1300
Glyceraldehyde-3-phosphate dehydrogenase	*cbbG*	Mfum_170031	1830	483	926
Fructose-bisphosphate aldolase	*fbaA*	Mfum_310038	2016	1688	1917
Transketolase (TK)	*cbbT*	Mfum_70010	2119	1527	2448
Protein CbxX	*CbxX*	Mfum_70008	6882	2807	8188
Ribulose bisphosphate carboxylase small subunit	*cbbS*	Mfum_70007	7200	3606	6693
Ribulose bisphosphate carboxylase large subunit)	*cbbL*	Mfum_70006	10209	7348	8567
RuBisCO operon transcriptional regulator CbbR	*lysR*	Mfum_140011	47	41	28
Carbonic anhydrase	*cynT*	Mfum_890009	1832	2022	851
Ribose-phosphate pyrophosphokinase	*prs*	Mfum_870060	1105	1091	1055

*The mRNA expression is expressed as RPKM according to Mortazavi et al. (2008). Changes in expression in the chemostat cultures (N_2_ fixing cells or O_2_ limited cells) compared to batch culture cells growing at μ_max_ are indicated by shading: up regulation >2 times (dark gray), down regulation <0.5 (light gray)*.

**Table A2:** **Transcription of genes involved the TCA cycle in “*Ca*. *M. fumariolicu**m*” strain SolV**.

Enzyme	Gene name	GenBank identifier	Expression level (RPKM)		
			Cells at μ_max_	N_2_ fixing cells	O_2_ limited cells
Pyruvate 2-oxoglutarate dehydrogenase complex (E1), alpha subunit	*acoA*	Mfum_180024	1016	891	813
Pyruvate 2-oxoglutarate dehydrogenase complex (E1), beta subunit	*acoB*	Mfum_180023	922	937	987
Pyruvate 2-oxoglutarate dehydrogenase complex, dihydrolipoamide acyltransferase (E2)	*aceF*	Mfum_180020	877	813	297
Pyruvate 2-oxoglutarate dehydrogenase complex, dihydrolipoamide dehydrogenase (E3)	*lpd1*	Mfum_180019	478	241	322
	*lpd 2*	Mfum_720053	121	42	103
Succinate dehydrogenase flavoprotein subunit	*sdhA*	Mfum_300009	419	359	256
Succinate dehydrogenase catalytic subunit	*sdhB*	Mfum_300008	177	84	231
Succinate dehydrogenase cytochrome *b* subunit	*sdhC*	Mfum_300010	185	72	59
Succinyl-CoA synthetase subunit beta	*sucC*	Mfum_170019	279	227	214
Succinyl-CoA ligase (ADP-forming) subunit alpha	*sucD*	Mfum_170018	831	1248	751
Pyruvate kinase	*pykF1*	Mfum_940089	155	86	130
	*pykF2*	Mfum_990021	630	382	499
6-Phosphofructokinase	*pfkA*	Mfum_920021	413	288	540
Deoxyribose-phosphate aldolase	*deoC*	Mfum_850005	100	44	92
Enolase	*eno*	Mfum_310014	1540	2423	1136
Fumarate hydratase class II	*fumC*	Mfum_220008	192	146	156
Phosphoenolpyruvate carboxykinase (ATP)	*pckA*	Mfum_200061	250	227	161
Isocitrate dehydrogenase (NADP)	*icd*	Mfum_200008	242	154	122
Aconitate hydratase	*acn*	Mfum_170037	343	170	156
Phosphoglycerate mutase (PhoE family)	*phoE*	Mfum_70011	530	282	306
Citrate synthase	*gltA*	Mfum_260030	830	404	368
Acyl-CoA synthetase (AMP forming)	*Acs*	Mfum_1020005	448	297	414
Acetate kinase	*ackA*	Mfum_310026	256	180	161

*The mRNA expression is expressed as RPKM according to Mortazavi et al. (2008). Changes in expression in the chemostat cultures (N_2_ fixing cells or O_2_ limited cells) compared to batch culture cells growing at μ_max_ are indicated by shading: up regulation >2 times (dark gray), down regulation <0.5 (light gray)*.

**Table A3:** **Transcription of genes involved carbon and energy storage in “*Ca. M. fumariolicu**m*” strain SolV**.

Enzyme	Gene name	GenBank identifier	Expression level (RPKM)		
			Cells at μ_max_	N_2_ fixing cells	O_2_ limited cells
Glycogen synthase 2	*glgA*	Mfum_1010040	306	242	346
1,4-Alpha-glucan-branching enzyme	*glgB*	Mfum_170041	154	91	110
Malto-oligosyltrehalose trehalohydrolase	*glgB*	Mfum_170046	162	128	81
Glucose-1-phosphate adenylyltransferase	*glgC*	Mfum_1020013	391	158	416
Glucan phosphorylase	*glgP1*	Mfum_1020098	656	447	843
	*glgP2*	Mfum_220010	113	98	122
	*glgP3*	Mfum_880004	447	382	450
Glycogen operon protein homolog	*glgX*	Mfum_40003	381	218	263
Glycogen debranching enzyme	*gdb*	Mfum_200059	256	246	106
ABC-type phosphate transport system, permease	*pstA*	Mfum_300005	81	45	98
Phosphate import ATP-binding protein	*pstB*	Mfum_300006	137	99	249
ABC-type phosphate transport system, permease	*pstC*	Mfum_300004	72	35	83
ABC-type phosphate transport system, periplasmic component	*pstS*	Mfum_300003	108	97	129
Exopolyphosphatase	*gppA1*	Mfum_1010048	581	430	486
	*gppA2*	Mfum_1020105	101	63	101
Polyphosphate kinase	*ppk*	Mfum_1030014	266	206	197
Exopolyphosphatase-related protein		Mfum_550017	506	283	404
Adenylate kinase	*adk*	Mfum_210014	247	88	170

*The mRNA expression is expressed as RPKM according to Mortazavi et al. (2008). Changes in expression in the chemostat cultures (N_2_ fixing cells or O_2_ limited cells) compared to batch culture cells growing at μ_max_ are indicated by shading: up regulation >2 times (dark gray), down regulation <0.5 (light gray)*.
